# Feasibility and Acceptability of Yoga for Adolescents with Juvenile Idiopathic Arthritis

**DOI:** 10.3390/children11070812

**Published:** 2024-07-02

**Authors:** Adina Dawoud, Jill Blitz, Steffany Moonaz, Leah Grout

**Affiliations:** 1 Division of Pediatric Rehabilitation Medicine, Children’s Hospital Los Angeles, Los Angeles, CA 90027, USAjblitz@chla.usc.edu (J.B.); 2 Department of Clinical and Health Sciences Research, Southern California University of Health Sciences, Whittier, CA 90604, USA

**Keywords:** adolescent, juvenile idiopathic arthritis, yoga, mind-body, integrative medicine, behavioral intervention, feasibility

## Abstract

Background/Objectives: Yoga is effective for adults with arthritis but unstudied in adolescents with juvenile idiopathic arthritis (JIA). Methods: A pilot study assessed the feasibility and acceptability of an 8-week group yoga intervention for adolescents ages 14–18 with JIA. Each 75-min session included breathing techniques, relaxation, mindfulness, and modified yoga postures, using yoga props and a rope wall. An online video was available for home practice. The outcome measures administered at the baseline and at 8 weeks were physician global assessment with joint count, visual assessment with a joint damage assessment index, the Pediatric Quality of Life Arthritis Module 3.0 (Peds QL), and the visual analog scale for pain. Results: Thirteen out of 25 participants attended ≥1 class with a mean of 5.7 ± 2.2 classes. Common reasons for non-enrollment included distance, schedule, and lack of interest. The average distance to classes was 29.0 ± 41.7 miles. There was a trend toward improvement for joint count (*p* = 0.07), global assessment (*p* = 0.10), and the Pain and Hurt domain of the Peds QL (*p* = 0.13), but no other outcomes approached significance. Satisfaction data from an anonymous survey (*n* = 8) were high in all areas. Conclusions: Adolescents with JIA who attended yoga reported enjoyment, pain reduction, and interest in continued practice with no adverse events. Future studies should consider stakeholder engagement to reduce barriers and larger sample sizes to test the effectiveness.

## 1. Introduction

Juvenile Idiopathic Arthritis (JIA) is a group of chronic systemic arthritis conditions with childhood onset. Beyond the effects of adult-onset arthritis, JIA can impact bone development, joint integrity, and overall growth [1]. Adolescents with JIA present with diverse disease symptoms, including joint swelling and stiffness, limited joint motion with morning stiffness, fatigue and limited endurance, muscle atrophy, secondary osteopenia secondary to long-term oral steroid use, and pain. These symptoms can lead to decreased quality of life and disease-related anxiety [2].

While similar in some respects to adult systemic conditions like rheumatoid arthritis (RA), unique challenges arise for the JIA population, including decreased activity levels and different psychosocial challenges than their healthy peers. Regardless of advances in medications, adolescents with juvenile idiopathic arthritis (JIA) are significantly less active than their peers, resulting in poor endurance, muscle weakness, obesity, and pain [1], which is highlighted as major barrier to physical activity for adolescents with JIA [3].

Despite the barriers, exercise is an important part of JIA management, as with all forms of arthritis. Exercise programs are beneficial for improving pain, range of motion, strength, function, and overall well-being in patients with JIA [4,5]. Moderate adherence to prescribed exercise was associated with reduced pain [4]. Physical activity was inversely related to pain interference, and it has been shown that physical activity interventions may play an important role in pain management in JIA [5]. Guidelines on exercise for JIA suggest that programs for adolescents should include postural and functional activities, strengthening, balance, stretching, proprioception, and aerobic activities [6,7,8].

Yoga may provide an accessible means to increasing physical activity by including the recommended components alongside relevant stress management strategies. A comprehensive yoga practice of movement, breathwork, relaxation, and meditation helped to improve functional ability, including balance and mobility, along with a reduction in pain and improved psychosocial outcomes in a diverse population of adults with RA and osteoarthritis (OA) [9]. Yoga has also demonstrated effectiveness for improving pain disability, mood, fatigue, pain acceptance, and self-efficacy in young adults with RA [10]. Other research has found that yoga participants with RA had improvements in pain along with decreased use of medications including corticosteroids, biologics, and NSAIDs [11]. Unfortunately, there have been no published clinical trials testing the effects of yoga for adolescents with JIA who could potentially benefit from the use of these practices for many years.

The purpose of this pilot study was to determine the feasibility and acceptability of an 8-week group yoga intervention for adolescents with JIA and its potential effects on pain and quality of life.

## 2. Materials and Methods

### 2.1. Participants

Participants included adolescents ages 14–18 years who could speak and understand English and get on and off of the floor independently. All participants were recruited from the Rheumatology Clinic at Children’s Hospital Los Angeles and were cleared by their attending rheumatologist prior to recruitment. Researchers were present in the rheumatology clinic for in-person recruitment. The exclusion criteria were any comorbidities contraindicated for physical activity or the use of an assistive device for ambulation. Participation was open to any interested patients regardless of their location if they were willing to travel and able to commit to the 8-week intervention.

### 2.2. Intervention

The intervention was delivered in two 8-week cohorts consisting of weekly in-person group yoga sessions for 75 min each, offered at a rented, off-site private yoga studio. Two cohorts were planned to ensure small class size, and each received the exact same intervention. The curriculum included breathing techniques and guided meditation, in addition to classical and adapted yoga postures with a final relaxation pose. A variety of standing, seated, supine, and prone poses were executed with the support of wall ropes, chairs, bolsters, blankets, and blocks. Some postures were hybrids of a classical yoga posture with a traditional exercise. The classes were designed to address range of motion, strengthening, balance, pain management, and breath regulation. The program was progressive and designed to build off of concepts and poses learned during the previous week. A variety of options were provided for each pose to accommodate the unique needs and limitations of each participant. For example, if a participant had limited wrist extension or pain in the wrists with weightbearing, the downward-facing dog pose was modified to use a wall strap and/or a chair to offset the weight load through the upper extremities (See Supplement Section S1).

The program was taught by a certified yoga therapist who is also a pediatric occupational therapist with more than 15 years of experience. A CHLA volunteer, trained by the teacher, assisted the teacher in class attendance and prop management but had no physical contact with the participants. Participants were provided a complimentary bus/METRO pass to cover round-trip transportation for the duration of the study. For families wishing to drive, parking was covered.

In addition to the group sessions, there was a 20-min online yoga video that the participants could access with a password for home practice between live classes. The video allowed participants to continue their yoga practice independently at home as frequently as they desired. It was professionally filmed with relaxation music composed by the videographer. The video consisted of grounding breathwork and asanas (postures) in supine and side-lying. Props such as a strap and block were utilized and were provided free of charge to all participants. The designed sequence was restorative in nature and included postures practiced in class that were familiar to participants.

### 2.3. Measurement

Safety was assessed through the reporting of adverse events, which were queried at each class and at times of data collection. Feasibility was measured through program participation, which included class attendance and visits to the home practice video. Acceptability was determined via an anonymous Likert-scale satisfaction survey, administered after the 8-week intervention, that included questions about the helpfulness of the program, the physical appropriateness of the classes, intention to implement recommendations provided during sessions, and the effect of the intervention on pain.

To determine the potential effectiveness of the intervention, clinical outcomes were measured at baseline and 8 weeks, assessing quality of life, pain, flexibility, and disease activity. After the first cohort, some outcome measures were changed, including the addition of a generic quality of life measure, the replacement of a pain scale for easier interpretation, and the addition of a fitness test as an objective measure of physical function (See Table 1). Altogether, these assessments took approximately 75 min and were scheduled immediately prior to the first yoga class and immediate after the last class at 8 weeks.

### 2.4. Data Analysis

Descriptive statistics (i.e., means and standard deviations) were used to summarize patient characteristics and patient outcomes for participants in both cohorts. Exploratory tests of significance were completed for outcome variables with 10 or more observations. Data for one patient were excluded from analyses because the patient experienced an unrelated disease flare, which resulted in missing data points and extreme outlier values for certain outcome variables. Histograms, Q–Q plots, and Shapiro–Wilk tests indicated that the differences between data pairs (i.e., pre- and post-scores) were normally distributed. Therefore, paired-sample *t*-tests were used to assess the differences in outcome measures before and after the yoga intervention (alpha level = 0.05). A sub-analysis by cohort was not possible due to the small sample size. Analyses were conducted in Excel^®^ for Microsoft 365 MSO (Version 2406) and R (v. 4.3.2).

## 3. Results

One hundred and eleven patients at CHLA meeting the criteria for participation were approached and informed about the study. Twenty-five consented to participate, including 18 for the first cohort (6 May 2017–24 June 2017) and 7 for the second cohort (30 August 2018–18 October 2018). Of those, 13 attended at least one class, with 10 in the first cohort and 3 in the second cohort.

### 3.1. Safety

There were no adverse events reported. One participant had a disease flare at the very end of the 8-week session, which was deemed unrelated to the intervention.

### 3.2. Feasibility

Less than a quarter of the patients who were informed about the study decided to enroll. Common reasons for non-enrollment of eligible patients included distance or transportation (*n* = 20), schedule conflicts or busyness (*n* = 11), expressed interest but did not provide consent (*n* = 10), unresponsive (*n* = 5), and lack of interest (*n* = 4). Others did not provide a reason. More than half of those who consented did not attend any classes. The reasons for non-participation were not recorded. For those who did participate in classes, the mean attendance was 5.7 ± 2.2 classes. Most participants (77%) attended at least five of eight classes. For those who consented, the average distance to the class location was 29.0 ± 41.7 miles with a range of 5 to 202 miles each way. Families reported traffic, driving distances, multiple children in the home, and the rigorous academic and extracurricular schedules for teenagers as limiting factors contributing to attendance. For the online yoga video, no views were logged. Families reported that they did not utilize the video because they forgot or did not have time.

### 3.3. Acceptability

Satisfaction data were available for eight participants related to pain improvement, program enjoyment, and likelihood of continuing yoga. The results of this survey are summarized in Table 2. With each item ranging from 1 (strongly disagree) to 5 (strongly agree), the median responses were 4 (agree) or 5 (strongly agree) across all items, indicating high levels of satisfaction and intervention acceptability for those who completed the survey.

### 3.4. Effectiveness

Data at both timepoints were available for the 13 participants who attended yoga sessions. The participant who experienced a flare toward the end of the intervention was removed from the analysis. The visual analog scale was completed by the first cohort only (*n* = 10). Items completed by the second cohort only (*n* = 3) are not reported due to small numbers (Wong–Baker Faces, Sit and Reach). The mean and standard deviations for each timepoint are reported in Table 3, along with *p*-values for the difference between timepoints for each item.

**Figure 1:**
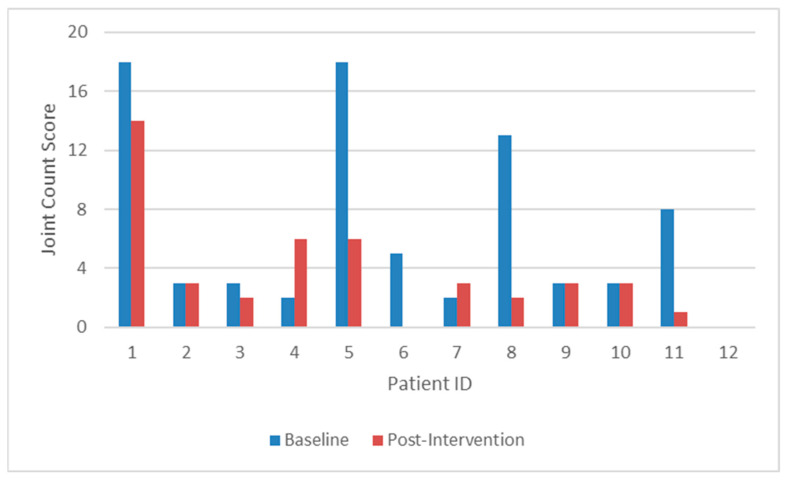
Changes in joint count scores from baseline to post-intervention (8 weeks).

Overall, joint count scores decreased by 12.03% (SD = 80.70) on average from the baseline to the end of the intervention (8 weeks). However, the observed changes were driven by larger changes for only a few participants. In total, there were six participants (6/12, 50%) with decreases in joint count scores, while four had no change, and two had an increase in joint count score from the baseline to the end of the intervention. The results of the exploratory paired-sample *t*-tests indicated that there was not a significant difference in joint count scores from the baseline (M = 6.50, SD = 6.35) to the end of the intervention (M = 3.58, SD = 3.80), t(11) = 2.04, *p* = 0.067. The changes in joint count scores for each participant from the baseline to the end of the intervention are shown in Figure 1. Similarly, global assessment scores increased by 18.69% (SD = 134.71), but there was not a significant difference in the scores from the baseline (M = 12.88, SD = 10.19) to the end of the intervention (M = 8.92, SD = 6.32), t(11) = 1.79, *p* = 0.101. Seven participants (7/12, 58.33%) saw a decrease in global assessment scores, two had no change, and three had an increase in global assessment scores from the baseline to the end of the intervention. While the other patient-reported outcomes also did not show a statistically significant change from the baseline to the end of the intervention, changes in the Pain and Hurt domain of the PedsQL suggested a trend toward improvement that might demonstrate significance with a larger sample size.

## 4. Discussion

To date there are no published trials reporting the feasibility or effectiveness of yoga for adolescents with JIA. The survey results showed that participants enjoyed the yoga program and reported less pain; however, challenges in recruitment and attrition led to a small sample size that does not allow for significant data results. Some measures demonstrated a statistical trend toward improvement that might reach significance with a larger sample. This suggests the need to examine factors that may contribute to these challenges and provide suggestions for improvement.

In terms of safety, there were no related adverse events, and there were improved disease symptoms, which indicate that a yoga intervention designed for adolescents with JIA is safe and warrants further investigation to determine effectiveness for improving disease symptoms. One participant did experience a disease flare during the intervention, which was attributed to the disease process by the treating rheumatologist and not to the yoga intervention. Yoga has been deemed safe via randomized controlled trials in adult rheumatic disease populations [9,10], and additional research is needed to confirm safety in JIA.

The qualitative survey results suggest that yoga was an acceptable and enjoyable workout for the participants. On a 5-point Likert, they reported enjoying the classes and experience. They also reported less pain and feeling better about themselves. This suggests a high level of acceptability among the survey completers who attended yoga classes. Due to the large variation in the symptoms experienced by patients with JIA, larger samples will be required to better understand the effect of yoga therapy on JIA. However, the preliminary data supports the fact that yoga may be helpful as a self-care strategy to help manage the challenges presented by JIA.

The reasons for not participating in the study varied among participants. Given that Children’s Hospital Los Angeles services the greater Los Angeles area and further, many patients travel up to two hours to see their rheumatologists, this led many participants to report that the program was too far away. The yoga studio was chosen because it was located near the hospital and in a central location. However, given the traffic patterns in Los Angeles, this still proved to be challenging for participants. Participants drove from all over Los Angeles and Southern California to participate in this specialized program that was unique to their child’s diagnosis. One participant drove three hours each way for this program because there was nothing similar available in her region. Everyone was given either a METRO pass or parking validation. Most chose to drive, and parking proved to be challenging even with the validation. Also, one phase was on a Saturday, whereas the second was on a Thursday after school times. Ten participants completed the sessions on Saturdays compared to only three on Thursdays. Future programs may want to first get input from the participants about convenient locations and times and perhaps consider offering yoga online to avoid travel concerns.

Another challenge among adolescents in general is their busy schedules. Often adolescents have a lot of homework, school obligations, and activities and do not have time for additional activities, despite their potential therapeutic value. This challenge was seen in other studies with adolescents as well [17].

As with any trial, this study has several limitations. First, the sample size is quite small. Challenges with study recruitment and retention provide important information about the challenges to feasibility but potentially bias our sample to those most willing and able to participate. While we have information about reasons for declining to participate, we are unable to report reasons for non-attendance among enrolled participants. One participant experienced a flare during the trial, and though it was deemed unrelated to the intervention by the participant’s rheumatologist and instead attributed to the disease process, the quasi-experimental study design makes it impossible to compare adverse events in yoga to rates by non-participants. Similarly, the quasi-experimental design leaves us unable to attribute any trends toward improvement to the yoga intervention. The satisfaction ratings are high, but the satisfaction survey was completed by a small proportion of participants, which limits its generalizability. In the satisfaction survey, participants reported the usefulness of the online program despite failing to use it. It is possible that the question was confusing to participants who thought that it was useful to include an online option even if they did not personally use it. Lastly, the measurement tools we used were changed between the cohorts. The generic module of the PedsQL was added, the pain scale was changed to the Wong–Baker Faces Scale for ease of use in a pediatric population, and the fitness test was added to gather data regarding changes in physical function. Because the second cohort was so small, there were insufficient data with these measures for inclusion in this report.

Given the importance of physical activity for long-term JIA management, it is essential to find opportunities that are safe and appropriate for those with chronic illness. Yoga has demonstrated effectiveness for improving disease symptoms and quality of life in adult rheumatic diseases [18,19,20], but it is relatively unstudied in JIA. As a mind-body practice, yoga also offers tools for stress management and cognitive reframing that can impact the whole-person experience of JIA, but general community-based yoga may not be appropriate for this population. While online yoga could reduce travel concerns, it would still have to consider schedule limitations if offered synchronously to allow direct instruction, supervision, and community support. The pre-recorded video was not viewed by any participants, which may suggest a lack of interest in asynchronous online content.

## 5. Conclusions

Adolescents with JIA who attended a population-specific 8-week yoga program reported enjoyment, pain reduction, and interest in continued practice. No adverse events were reported. This suggests that yoga may be safe and acceptable for this population. A trend toward improvement in joint count, global assessment, and Pain and Hurt domains suggest possible effectiveness for improving the clinical and patient concerns that must be studied with larger samples. These findings must be interpreted with great caution due to the very low enrollment and attendance, which indicate barriers to feasibility such as scheduling and distance. Future studies should consider stakeholder engagement through surveys and/or focus groups to understand and reduce barriers to participation in this promising intervention.

## Figures and Tables

**Table 1:** Outcome measures administered by the cohort for yoga participants with JIA.

Instrument	Format	Description	Scoring	Cohort 1	Cohort 2
Pediatric Quality of Life Inventory (Peds QL)—Generic Core Form 4.0 [12]	Patient-reported outcome	One-page questionnaire with questions regarding health and activities (eight items), feelings (five items), getting along with others (five) and school (five)	Likert scale of 0–4. Scores range from 0: “never” to 4: “almost always” a problem with daily life.		X
Pediatric Quality of Life Inventory (Peds QL)—Arthritis Module 3.0 [13]	Patient-reported outcome	One-page questionnaire assessing perception of pain and hurt (four items), daily activities (five items), treatment (seven items), worry (three items) and communication (three items)	Likert scale of 0–4. Scores range from 0: “never” to 4: “almost always” a problem with daily life.	X	X
Visual Analog Pain Scale	Patient-reported outcome	14 cm line with 0 as no pain and 14 for pain as bad as it could be	Scored 0–14 by measurement in centimeters	X	
Wong–Baker FACES Pain Rating Scale [14]	Patient-reported outcome	The scale shows a series of faces ranging from a happy face which represents “no hurt” to a crying face which represents “hurts worst.” Valid and reliable for ages 3–18.	Scores range from 0 (no hurt) to 10 (hurts worst).		X
Sit and Reach Flexibility Box [15]	Fitness testing	A measure used to assess flexibility of the lower back and hamstring muscles. It involves stretching out toward the toes or beyond with extended arms from sitting position.	A yardstick was used for measurement with 15 inches at the toes with average scores for adolescents ranging from 8–12 inches, varying by age and sex.		X
Juvenile Arthritis Damage Index—Articular (JADI-A) [16]	Clinical assessment	Damage is scored for each listed joint when present for at least 6 months, explained by prior damage and not due to current arthritis activity.	Maximum score of 72. A higher score indicates more involved joints and/or a greater degree of damage.	X	X
Juvenile Arthritis Damage Index—Extraarticular (JADI-E) [16]	Clinical assessment	Assesses persistent changes in anatomy, physiology, pathology or function, persisting for at least 6 months.	Maximum score of 17. A higher score indicates more system involvement and/or greater severity.	X	X
Physician Global Assessment of Disease Activity (PhGA) [16]	Clinical assessment	Captures examiner’s subjective appraisal of the patient’s disease activity at time of visit.	Visual analogue scale (VAS), with anchors of 0 for no activity and 100 for maximum activity	X	X

**Table 2:** Median and interquartile range (IQR) of responses to satisfaction survey items.

Item	Median (IQR)
The program was fun.	5.0 (0.0)
The program was at a level that I could follow.	5.0 (0.0)
I found the online program useful.	4.0 (1.0)
I feel that I will continue with yoga now that the program is over.	4.0 (1.5)
I feel that my pain has decreased since starting this yoga program.	4.0 (0.0)
I feel better about myself since starting this yoga program.	4.0 (1.5)

**Table 3:** Patient outcomes measured prior to and following the 8 weeks of yoga therapy.

Outcome	*n*	Before Yoga Mean (SD)	After Yoga Mean (SD)	*p*-Value
Visual Analog Pain Scale	10	3.67 (3.45)	3.71 (2.74)	0.96
Peds QL: Pain and Hurt	12	61.82 (20.50)	68.78 (19.41)	0.13
Peds QL: Daily Activities	12	83.75 (18.12)	82.08 (12.70)	0.65
Peds QL: Treatment	12	74.70 (14.56)	78.28 (13.83)	0.49
Peds QL: Worry	12	57.64 (25.50)	51.39 (30.33)	0.25
Peds QL: Communication	12	66.67 (18.81)	64.58 (21.64)	0.70
JADI-AD	12	0.17 (0.58)	0.17 (0.58)	1.00
JADI-ED	12	0.00 (0.00)	0.00 (0.00)	1.00
JADI Joint Count	12	6.50 (6.35)	3.58 (3.80)	0.07
Global Assessment	12	12.88 (10.19)	8.92 (6.32)	0.10

## Data Availability

Deidentified data that support the findings of this study are available from the corresponding author upon reasonable request. The study data are not publicly available due to ethical reasons.

## Supplementary Materials

The following supporting information can be downloaded at https://www.mdpi.com/article/10.3390/children11070812/s1: Section S1. Class Structure. Section S2. Class Sequences. Section S3. Sample Posture Descriptions.
