# Supplementary material for: Feasibility and Acceptability of Yoga for Adolescents with Juvenile Idiopathic Arthritis
Source: Children (Basel). 2024 Jul 2;11(7):812. doi: 10.3390/children11070812 (PMC11276325; doi:10.3390/children11070812)
Supplement: Supplementary file 1 [file children-11-00812-s001.zip › children-3016876-supplementary.pdf]

Section S1 Class Structure

Class set up and check-in: 5 minutes

- Instructor and volunteer set up each participants yoga space with all props needed for class.
- Participants checked in with teacher individually if there were any issues of concern (pain, menstruation, fatigue) to report.

Breathwork: 5 minutes

- Diaphragmatic breathing (3-part breathing), modifications with gentle pauses
- Ujjai (ocen breath) traditional yoga breathing through nostrils
- Breath work in supine with blanket on belly; or breathwork in sukasana

Postures: 55 minutes

- Traditional postures modified to use the rope wall, chairs, blocks, straps and blankets.
- Postures included: standing, seated, twists, supine
    - 10 minutes warm up of floor postures increasing body awareness and build heat
    - 20 minutes full body standing postures, including twists
    - 10 minutes therapeutic strengthening hybrid postures for specific body parts
    - 15 minutes seated postures, cool down

Final Relaxation/Corpse pose: 10 minutes

- 5 minutes: Guided relaxation and natural breathing in supported postures
- Supported postures included: supported savasana with blankets under knees and head in supine; Viparita Karani (legs up wall) or modified with calves on chair while in supine; supported supta baddha konasana with blanket rolls under knees, blanket under head
- 5 minutes: Savasana/corpse pose

Section S2 Class Sequences

Class 1

- Breath work in supine, bolster under knees (blanket on abdomen for feedback)
- Supine Apanasana / knee to chest
    - Focus on work of standing leg and abdomen sinking→ twist
- Supine twist (block between knees)
- Puppy Dog (back of chair @ wall, feet wide)
- Utthita Uttanasana: chair twist (standing, legs wider than hips, one arm on chair back)
- "Superwoman arms" with rope x10 (full shoulder range of motion into back stroke arms)
- Trikonasana (rope around hips, heel @ wall, hand on chair)
- Ardha Chandrasana (heel @ hook for support, forearm on chair)
- Tree (prep balance with alt. hip flexion with arm support at wall; advanced option: stand on bolster)
- Supta Badha Konasana (bolster, strap, blanket x2)
- Savasana (calves on chair)

Class 2

- Breath work on back with calves on chair (blanket on abdomen for feedback)
- Supine Apanasana / knee to chest
    - Focus on work of standing leg and abdomen sinking→twist
- Supta Padangusthasana A & B (heels at wall, strap)
- Puppy Dog "push wall" A) feet mats' width; B) feet hip width
    - If wrist pain or limited range of motion, use chair
- Vrksasana (Tree pose) (Options: on bolster, foam block on head for balance challenge)
- Warrior II (Viravadrasana II) with heel at wall and back arm at wall
- Utkatasana on chair
- Garudasana (Eagle)
    - On chair teach legs with arms
    - Standing full pose as option or remain in chair
- Setu Bandha (Bridge pose) Restorative with blankets or blocks under sacrum
- Paschimmotanasana with feet at wall; emphasis on length in spine; can put chair over legs to reach for chair seat to prevent back rounding
- Savasana supported with props/bolster under knees

Class 3

- Balasana: Breath work in child's pose (blankets for forehead and under glutes)
- Bird dog; alternate arm/leg extension or in supine with hip flexion as alternative to weightbearing
- Sukasana on blanket
    - Twist; alternate sides with opposite leg in front
- Bhujangasana/Sphinx hybrid dynamic with blanket slides

- Salabasana (modified with alternating arms/legs)
- Puppy Dog "push wall" A) feet mats' width; B) feet hip width
    - If wrist pain, use chair
- Warrior I (Viravadrasana I) with back heel at wall
  Warrior III (arms reaching forward to chair, option)
- Navasana/modified with feet on floor and hands touching knees, alternating reaching arms into flexion
- Dandasana with back at wall (hands on chair seat if necessary)
- Savasana supported with props/bolster under knees

Class 4

- Breathwork supine with bolster and blanket in cross shape for chest opening
- Thread the needle with feet at wall for support/feedback
- Sukasana on blanket
    - Twist; alternate sides with opposite leg in front
- "Superwoman arms" with rope x10 (full shoulder range of motion into back stroke arms)
- Puppy dog at wall or Adho mukha svanasana with hands on chair seat or on floor with two blocks as lifts (if able)
- Use Strap for "parachute arms"-chest opening → Arm/chest/pec stretch standing at wall with arm at wall at 90* shoulder abduction, stepping counterclockwise.
- Utthita Parsvakonasana with supporting forearm or hand on thigh
- Gomukasana (arms with strap)
- Supported Setu Bandha (blocks under hips)
- Viparita Karani (blanket under hips, legs up wall) as Savasana (or standard supine)

Class 5

- Breathwork seated in sukasana on blanket
- Seated sukasana lateral side bend (seated on bolster or blanket if necessary, grounding opposite hip)
- Supta tadasana on floor with shoulder flexion (learning actions of legs and arms)
- Supta padangustashana with strap
    - Padangustashana standing with foot supported on chair and arms in shoulder flexion
- Navasana/modified with feet on floor and hands touching knees, alternating reaching arms into flexion
- Puppy dog at wall, legs hip width (chair if needed)
- Utthita Marichyasana at wall with foot on chair
- Supta baddha konasana
- Savasana with shins on chair

Class 6

- Breathwork in supine, blanket roll vertical for spine/ chest opener

- Supta Padangustasana with strap A and B with bolster at hip
- Puppy dog with chair
- Parsvotonasana with chair
- Garudasana with chair→without chair butt at wall
- Warrior I with chair (option to add Garudasana arms)
- Arm flossing/nerve stretch & “back stroke arms”-blanket “tootsie roll” under feet to stretch plantar fascia
- Utthita Marichyasana at wall with foot on chair
- Puppy Dog with chair
- Vrksasana (option: feet on bolster)
- Upavishta Konasana towards chair seated on blanket
- Savasana with calves on chair or Viparita Karani up the wall

Class 7

- Viparita Karani, breath work/opening
- Supta Padangustasana A (strap)
- Gate Pose
- Balasana with hands on blocks 3 ways/heights for shoulder opening (forehead on blanket)
- Puppy Dog (chair if needed)
- Parsvotanasana w chair (back heel on blanket for tight calves)
- Bhujangasana/Sphinx hybrid dynamic with blanket slides
- Setu Bandha (Supported with block, legs strapped, shoulders on blanket, feet on block at wall)
- Janu Sirsasana
- Savasana (bolster or blanket under knees)

Class 8

- Viparita Karani , or calves on chair, breath work
- Supta tadasana-arms extended overhead thumb lock
- Bananasana (Yin pose)
- Gate Pose
- Downward dog with rope at pelvis and hands on chair
- Parsvotonasana with chair
- Bhujangasana/Sphinx hybrid dyanmimc with blanket slides
- Salabasana-alternate arm leg, option for full pose
- Supine apanasana (alternate legs)
- Supta Badha Konasana
- Savasana (bolster or blanket under knees)

Section S3 Sample Pose Descriptions

Names of Postures	Image with Modifications	Description of Modifications
Adho Mukha Svanasana	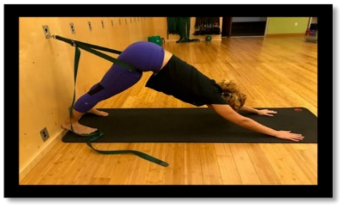	Use of rope wall with this pose removes the heavy weight bearing element from upper extremities. It allows participant to successfully engage in posture with weight supported. Heels at wall for feedback into legs for active engagement.
Modification for Puppy Dog	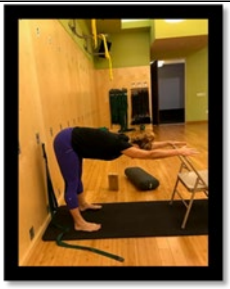	For participants who do not have full wrist extension for pose at wall, use of a chair allows full engagement with wrists in neutral and no weightbearing.
Utthita Matsyendrasana	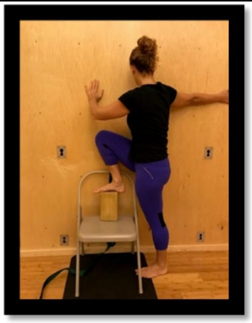	Modification of a seated twist in standing to allow participants to learn the components of a twist and teach the torso how to twist with supports and feedback of wall.
"Parachute Arms" with strap	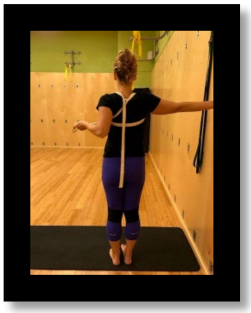	Use of strap teaches enhanced postural engagement of shoulders externally rotated. Use of opposite hand at wall for pectoralis stretch.
Utthita Trikonasana	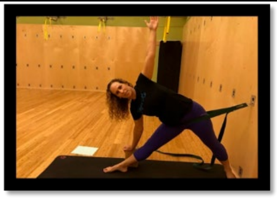	Use of wall rope to assist in supporting body weight to allow hamstring stretching and torso elongation.

Ardha Chandrasana	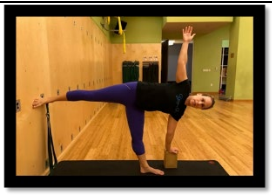	Use of wall for feedback and balance support to keep top leg engaged, torso elongated, and weightbearing leg externally rotated.
Modified Uttanasana with Twist	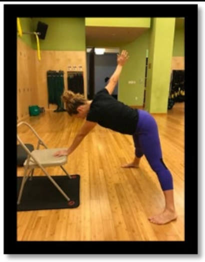	Use of chair allows participant to work on hamstring lengthening and torso twisting at a modified intensity.
Modified Sphinx	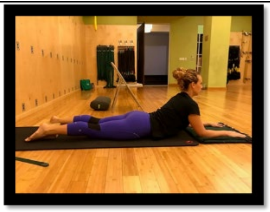	Use of blanket under forearms to provide dynamic strengthening to posterior chain muscles by sliding blanket forward and back.
